# Can primary mental health services impact levels of involuntary admissions? A cluster-RCT of the ReCoN intervention

**DOI:** 10.1007/s00127-025-02914-3

**Published:** 2025-04-30

**Authors:** Jorun Rugkåsa, Olav Nyttingnes, Jūratė Šaltytė Benth, Tonje Lossius Husum, Solveig Helene Høymork Kjus, Irene Wormdahl, Tore Hofstad, Trond Hatling

**Affiliations:** 1 Health Services Research Unit, Akershus University Hospital, Lørenskog, Norway; 2 Faculty of Health Sciences, Oslo Metropolitan University, Oslo, Norway; 3 Centre for Care Research, University of South-Eastern Norway, Porsgrunn, Norway; 4 Centre for Population Health, Haukeland University Hospital, Bergen, Norway; 5 Institute of Clinical Medicine, Campus Ahus, University of Oslo, Oslo, Norway; 6 Norwegian Resource Center for Community Mental Health, NTNU Social Research, Trondheim, Norway; 7 Department of Mental Health Work, NTNU Social Research, Trondheim, Norway

**Keywords:** Involuntary psychiatric admissions, Coercion reduction, Primary mental health care

## Abstract

**Purpose:**

Internationally, policies and legal changes seek to reduce the use of involuntary psychiatric admissions. Usually directed towards specialist services, these initiatives show little sustained progress. We tested whether an intervention at the level of primary mental health services has potential to reduce the use of involuntary admissions.

**Methods:**

We conducted a two-arm cluster-RCT following Zelen’s design (ClinicalTrials.gov:NCT03989765). Each cluster included the primary mental health service and their local collaborators in mid-sized Norwegian municipalities with rates of involuntary admissions above the national average. Five clusters were randomised to co-create and implement a comprehensive intervention. These could not be blinded, but the five control clusters were. Our primary hypothesis was that rates of involuntary admissions would be lower in the intervention arm when comparing change over time between arms, and that this would sustain. Secondary hypotheses were that rates of referrals for involuntary admissions and rates of referrals confirmed for involuntary status after the second statutory assessment, would be lower in the intervention arm.

**Results:**

Data obtained from the Norwegian Patient Registry included all events in the study period. The difference between trial arms in changes of rates of involuntary admissions from baseline to the intervention period was 6.8 (95% CI 1.8 to 11.7; effect size (EC) 2.7), and reduced to 3.0 (95% CI -3.8 to 9.7; ES 0.9) between baseline and the post-intervention period. The difference between arms regarding changes in referral rates between the baseline and intervention period was 1.7 (95% CI -4.6 to 8.1; ES 0.5), and for changes in the rate of referrals resulting in involuntary status it was 1.3 (95% CI -3.4 to 6.0; ES 0.8).

**Conclusion:**

We found a clear difference between trial arms in our primary outcome of involuntary admissions during the intervention period, but not beyond that period, and not regarding referrals for involuntary admissions, although the consistent direction of change favoured the intervention. We interpret the results to constitute ‘proof of concept’ that adequately resourced primary mental health services might contribute to policy aims of reducing involuntary care. Further rigorous studies in heterogeneous contexts are required.

**Supplementary Information:**

The online version contains supplementary material available at 10.1007/s00127-025-02914-3.

## Introduction

Despite a range of policies to reduce the use of coercion [1], population rates of involuntary psychiatric admissions are increasing in many countries [2]. There is criticism that the continuous closing of inpatient beds is not adequately compensated for by building up capacity in the community [3], and that this mismatch pushes compulsion rates up [4, 5]. Nonetheless, mental health care systems across western countries predominantly move towards community-based care. This means that those using services due to severe mental health problems typically do so while living at home and that signs of deterioration are detected in the community setting. Referrals to involuntary care are often made by general practitioners (GP) or out of hours emergency doctors (A&E) [6]. Processes that ultimately lead to involuntary admissions therefore typically take place before the person is referred to, or arrive at, the hospital [7]. Even so, initiatives to reduce involuntary admissions are usually directed towards specialist services, that is Health Trusts or hospitals with legal authority to establish and maintain involuntary care [8, 9].

Systematic reviews show that various interventions to reduce the use of inpatient coercive measures (e.g., seclusion and restraint) have positive results [10, 11]. Due to the complexities involved, interventions with multiple components are recommended [10, 12]. As regards community-based interventions to reduce compulsion, individual studies of initiatives to prevent or plan for crises (e.g., joint crisis plans, advance statement, shared decision making, risk assessments and staff training) show promising results [13], but reviews conclude that the literature is too limited in size and quality to draw conclusions [1, 11]. This is also the case with more complex interventions such as community crisis residential care or various crisis resolution initiatives [14]. Community Treatment Orders (CTOs), legal powers enabling community compulsion with the aim to reduce (re)admissions, have a stronger evidence base. A recent meta-analysis concluded that CTOs do not lead to reduction in admission [15], but the included studies did not distinguish between voluntary and involuntary admissions. Assertive Community Treatment and Flexible ACT are comprehensive interventions found to improve follow-up and continuity of care in the community [16, 17], and potentially reduce involuntary care [18]. However, these service models are also wholly or partly organised by specialist services and not available to all.

Gooding and colleagues argue that rather than distinguishing between inpatient and outpatient interventions to reduce involuntary admissions, it would be more in line with current practice to distinguish between acute/crisis oriented vs. general support services. By general support they mean “*the range of non-urgent community-based services that currently exist to help prevent emergencies and assist people to live full lives”* [14, p 116]. Several review studies argue that such services, when combined with provision for crisis resolution, could potentially reduce the need for involuntary admissions [11, 14, 19]. We conducted a systematic literature search (search terms in supplementary material), but found no intervention developed for generalist primary mental health services with the aim to prevent involuntary admissions. As we detail below, Norwegian local authorities offer general mental health support, without specialist referral, to its citizens in need. This makes Norway a fruitful context in which to investigate whether an intervention at this service level might affect the pathways of those at risk of involuntary admissions. In the first stage of our study we co-created such an intervention together with local stakeholders [20]. The second stage, which we report here, tested the resulting Reducing Coercion in Norway (ReCoN) intervention. Our primary hypotheses were that, compared to baseline in each arm, there would be (i) larger change, with reduced rates of involuntary admissions in the intervention arm between the baseline and intervention periods compared with the control arm, and (ii) that this difference would sustain beyond that period. Secondary hypotheses were that changes in (iii) rates of referrals for involuntary admission and (iv) rates of such referrals being confirmed for involuntary status following the statutory second assessment, would see larger change with reduced rates in the intervention arm than the control arm during the intervention period compared with the baseline period.

## Methods

### Design

The service-level intervention precluded randomisation of individuals. We therefore conducted a pre-registered, two-arm cluster-RCT (ClinicalTrials.gov: NCT03989765). Clusters consisted of municipal primary mental health services and their local collaborating services and user/carer organisations. As we tested a new intervention, co-created and implemented by the intervention clusters, with no activity in the control arm and a finite number of eligible clusters, we applied a Zelen’s single consent design [21, 22]. Control clusters represented ‘treatment as usual’. The test of effect compared changes in outcomes over time at cluster level during the study period Oct 1, 2019 to Sept 30, 2022. The municipalities randomised to intervention that declined participation were not taking part in co-creating or implementing the intervention, so no patients here could be exposed to it. An intention-to-treat (ITT) analysis, which is recommended for Zelen’s design [22], would therefore not be meaningful, and would risk diluting potential effects. Our analyses were therefore per protocol. Data were obtained directly from the Norwegian Patient Registry (NPR), to which it is mandatory to report all specialist service contact [23]. As we obtained permission to obtain data without individual consent, complete data on all involuntary admissions in both arms were included. This also eliminated the issue of ‘non-completers’.

### Study context and legislation

Primary care, including mental health care, is in Norway the responsibility of local authorities, which include 356 municipalities and 15 city boroughs in Oslo (hereafter ‘municipalities’). These differ in total population size between 200 and 270,000. There have been substantial reductions in bed numbers and scaling up of community based mental health care in Norway over the last three decades [24]. Municipal multi-disciplinary primary mental health & addiction services follow up people with mental health problems at home, often for many years. This includes those at risk of involuntary admission, some of whom are subject to CTOs. Follow-up can include day-care facilities, handling/administrating medication, psychotherapeutic treatment and support, transport to GP or other appointments, support with shopping, nutrition or household chores, provision of or assistance to partake in leisure or social activities, and supported housing with or without resident staff. Municipalities must also ensure that its inhabitants have access to GP and A&E services, and they administer social care, social security benefits, employment services and social housing.

Specialist mental health care is the responsibility of four Regional Health Authorities, through 20 General Hospital Trusts with acute and high security psychiatric wards, and Community Mental Health Centres (CMHC) that provide specialist community-based inpatient and outpatient services. Municipalities are nested within catchment areas of CMHCs, of which there were 77 in the study period, and the two organisational levels are expected to work closely together [25]. Specialist services administer the Norwegian Mental Health Act (1999) that, given the criteria set out in Box 1 are met, permits involuntary admission (or CTO if deemed in the person’s best interest) for either observation or care.

**Box 1:** Criteria for involuntary care under the Norwegian Mental Health Act

Involuntary admissions can be for up to 10 days for observation purposes (Sect. 3.2) or for three-month, renewable periods for treatment (Sect. 3.3), on an inpatient or outpatient (CTO) basis if the following criteria are fulfilled:
• The person has a serious mental disorder (or in the case of observation, highly likely to have one)
• Voluntary care has been tried or is obviously futile
• The person has been examined by two physicians, one of whom is independent of the institution
• The person lacks capacity to consent to treatment (does not apply if there is serious risk)
• Involuntary care is necessary to prevent the person from deteriorating or having reduced prospects for getting better, or
• The person constitutes an imminent and serious risk to their own life or the health or life of others
• The institution is professionally and materially capable of offering satisfactory treatment
• The person has been given the opportunity to state his or her opinion
• Involuntary care is overall considered the best option for the person concerned

When deterioration is detected (often by the person in question, a family member or a municipal service provider), a medical doctor, typically a GP or A&E doctor, first assesses whether an involuntary admission is needed. If referred for an involuntary admission, the person is transported, sometimes by the police, to an acute hospital ward. Here, a second clinical assessment must confirm within 24 h that the legal criteria are (still) met. In 38% of cases the initial assessment is not confirmed [9]. Those on CTOs can have their order converted to inpatient involuntary status without referral.

### Cluster inclusion criteria and sample size considerations

Because we tested a new intervention, we wanted to ensure a degree of homogeneity among clusters to aid interpretation of results. We restricted therefore inclusion to mid-sized municipalities with a total population of 20,000–50,000 in 2018. To avoid potential floor effects, we included municipalities with rates of involuntary admissions above the national average rate per 10 K population 18 years or older. We determined this by averaging rates for the last two years of available data (2016 and 2017) to reduce the impact of random fluctuation. To reduce spill-over effects we did not include neighbouring municipalities, and excluded those with lowest rates. Should a municipality decline participation, however, their neighbour would no longer be considered ineligible: this procedure was to reduce the span in the involuntary admissions rates in the sample. We excluded municipalities affected by the national reorganisation of municipal boundaries that was underway at the time, because this could involve substantial restructuring of services during the intervention period.

Given the complexity of the co-creation process [20], it was necessary to keep the number of clusters at a manageable size. We expected that outcome data would only be available aggregated to the municipality level, which precluded power calculations at the individual level. Based on available statistics, we assessed that a mid-sized municipality would have 40–100 involuntary admissions each year. We included clusters with high rates, but based our calculations on the conservative estimate that five intervention and five control clusters would comprise at least 200 admissions in each arm. Assuming a significance level of 5%, this would give power well above 90% to detect between-group difference of 15% in change in involuntary admissions and with sufficient power preserved also when considering the primary measure over two time periods, corresponding to a significance level of 2.5%.

### Randomisation and masking

All municipalities that met the inclusion criteria (*n* = 28) were ranked in descending order of involuntary admission rates. We applied paired stratification, by which the first municipality on the list was paired with the second, number 3 with number 4, and so on. Starting with the highest ranking pair, one municipality was drawn at random to the intervention arm, and the other became control. We then invited the municipality randomised to intervention to take part. Should a municipality decline, the pair was excluded. The randomisation was conducted by an independent colleague, and continued until we had five included pairs. While controls were masked, it was not possible to mask professionals in the intervention arm or user/carer organisations involved.

### Procedures

In the intervention arm, the full range of stakeholders involved in processes leading up to someone being admitted involuntarily participated in co-producing the intervention in the period February-June 2020, as described in full elsewhere [20]. This was based on our mapping (during Autumn 2019) of pathways typically ending in involuntary admissions through qualitative interviews with 103 stakeholders, including people with severe mental health problems, some of whom had experienced involuntary care; family carers; municipal and specialist mental health professionals and managers; GPs, A&E doctors and chief municipal medical officers, and; police officers. Results [7, 26] were presented at dialogue conferences [27] that involved representatives from the same stakeholder groups. The purpose was to identify measures by which municipal mental health services, together with the other local stakeholders, could improve pathways within existing resources. As comprehensive interventions are recommended [10, 19], a wide range of measures were discussed, prioritised and taken forward in a set of iterative discussions between key stakeholders and the research team, resulting in the ReCoN intervention.

As shown in Box 2, the intervention consists of six strategy areas, each of which contains several areas of action, which again have a number of specified action points, 53 in total [20, 28]. These were considered potentially to improve support structures; enhance user involvement, and; strengthen skills, competencies and collaborations. Local municipal mental health services led the implementation in their area. They were supported by the research team who organised regular ‘trouble shooing meetings’ (10 with professionals and 10 with local user/carer organisations) and training courses (4 in total) during the intervention period, but not in the post-intervention period. Details of the nature and scope of the implementation support and structure is available elsewhere [28]. The implementation was conducted by, and as part of, existing services, with no additional staff or resources provided. The implementation evaluation found that the approach was feasible and that most actions were realised. Measured qualitatively in regular progress interviews at each site (45 in total), we found that the degree of implementation of the actions varied between 73 to 95% across sites, and there were no clear patterns of difference between strategy areas, except some difficulties with collaboration across service levels, partly due to Covid-19 restrictions [28]. No project activity took place in the control clusters, which continued to provide services ‘as usual’, as outlined in the description of study context above.

**Box 2:** The RECON-intervention*

**Strategy area 1: Management**
1.1 Management anchoring of the intervention in the collaborating services
1.2 Data monitoring – use of data of those admitted and success stories in service development
1.3 Continuous focus on service improvement
**Strategy area 2: Involving Persons with Lived Experience and Family Carers**
2.1 Involving persons with lived experience and family carers at the organisational level of the intervention
2.2 Adopting/maintaining post-incident reviews
2.3 Adopting/maintaining joint crisis plans
2.4 Having peer workers among staff
**Strategy area 3: Competence Development**
3.1 Adopting/maintaining a recovery-oriented framework
3.2 Accessing competence-building training courses
**Strategy area 4: Collaboration across Primary and Specialist Care Levels**
4.1 Collaboration when assessing someone for involuntary admission
4.2 Collaboration during and following involuntary admissions
4.3 Establishing/maintaining joint meeting points
**Strategy area 5: Collaboration within the Primary Care Level**
5.1 Structured collaboration between GPs/A&E services and the primary mental health services
5.2 Establishing/maintaining joint meeting points
**Strategy area 6: Tailoring Individual Services**
6.1 Focus on individually tailored accommodation
6.2 Develop primary level crisis or short-term placement
6.3 Provide support for meaningful everyday lives

*Details of the in total 53 specified action points is available elsewhere [20]

### Primary and secondary outcome measures

We measured outcomes over three time periods. The baseline period was from Oct 1, 2019 to Sept 30, 2020, the intervention period from Oct 1, 2020 to March 31, 2022, and the post-intervention period from April 1 to Sept 30, 2022. Our primary outcome measures were changes in incidence rates of involuntary admissions between (i) the baseline and intervention periods, and (ii) between the baseline and post-intervention periods. We also report on secondary outcome measures including (iii) changes in incidence rates of referral for involuntary care, and (iv) changes in the incidence rates of referrals confirmed for involuntary status, between the baseline and intervention periods.

### Data and variables

All outcome data were provided directly from the NPR with no involvement of services. We received disaggregated data on all events of involuntary admissions and all referrals for such admissions, for those 18 years and older in the 10 municipalities during the study period. In addition to dates on these events, and whether or not referrals ended in involuntary status, we obtained information as recorded in the registry on gender (male/female), age (in years), diagnosis (International Classification of Diseases-10 codes), and resident municipality associated with each event.

We used the software R to construct variables for estimating outcomes. Counts of *involuntary admissions* include all new events of admissions under the Mental Health Act for observation or treatment in the period. In a small number of cases, the time of arrival and formal decision differed with more than 24 h, possibly due to clerical or procedural inaccuracies. We included admissions with formal decisions dated two days before and up to seven days after arrival date, as we considered this related to the event and therefore to circumstances in the community. This means the count included a small number of cases where a voluntary admission was converted to involuntary within this timeframe. CTOs revoked to involuntary admissions were included. Because we counted events, a person could have more than one admission during a time period.

Counts of *referrals for involuntary admission* include all registered referrals for involuntary observation or treatment, whether or not they resulted in such admissions following the second clinical assessment. The unusual event of someone on CTO being formally referred because they presented to a service without access to their records were counted: the NPR does not include information on CTO endpoints so this group could not reliably be identified.

*Referrals confirmed for involuntary status* was a binary measure indicating whether or not the second assessment confirmed involuntary status (involuntary admission or the establishment of a CTO) within seven days.

Incidence rates were calculated using the annual population size (2019–2022) of those 18 years and older in each municipality. These data are publicly available at Statistic Norway’s website.

### Changes to protocol

We originally planned for a 12-month intervention period. At the request of the intervention clusters, who experienced that the Covid-19 pandemic slowed down implementation [28], this was prolonged to 18 months, and the post-intervention period consequently shortened to 6 months. Our registered protocol stated erroneously that we would measure prevalence, and not incidence rates of events per capita, which is the appropriate measure. Due to constraints in the data, it was not feasible to calculate duration of involuntary admissions, which originally was a secondary outcome. Following the qualitative process evaluation [28], we conducted a post-hoc explorative sub-group analysis of possible effects of the degree of implementation on the primary outcome. This showed stronger effect in low-implementation clusters, but qualitative measurements and the lack of established fidelity or implementation tools meant these results were difficult to interpret and potentially biased. They are therefore not shown, but available on request.

### Statistical analyses

In each time period, incidence rate in the corresponding arm was calculated as weighted sum $\:\stackrel{-}{p}=\sum\:_{i=1}^{5}{w}_{i}{p}_{i}$ with a variance $\:var\left(p\right)=\sum\:_{i=1}^{5}{w}_{i}^{2}{p}_{i}(1-{p}_{i})/{n}_{i}$. Here $\:{p}_{i}$ is a rate within municipality *i* and $\:{w}_{i}=\frac{{n}_{i}}{N}$, where *N* is total number of inhabitants within each arm. We present these rates per 10 K adult per 12 months (as the time periods varied in length) with 95% confidence intervals (CIs), defined as $\:\stackrel{-}{p}\mp\:1.96\sqrt{var\left(p\right).\:}\:$ The differences in rates between arms is presented with 95% CI calculated as above, but substituting $\:var\left(p\right)$ with a sum of variances in the two arms. For all outcomes, the differences between the intervention and control arm in change between specified time periods were assessed by z-test for proportions. We also present effect size (ES, Cohen’s d). All 10 clusters were included in all analyses.

We conducted two sets of pre-specified sensitivity analyses, using the same approach as for the main analyses. First, as our concern was involuntary admissions due to severe mental health problems, we repeated the analyses including only those aged 18 to 65 to avoid admissions related to dementia or other age related factors potentially affecting results. Second, we analysed admissions for observation and treatment separately, as these two forms could potentially be affected differently by the intervention.

For all outcomes, we assessed possible cluster effects at municipality level by calculating intra-class correlation coefficients (ICC) prior to aggregating the data. These effects were between 1.6 and 2.1%, so we considered them negligible. Analyses were performed in STATA v.17. Results with p-values below 0.05 were considered statistically significant. No adjustments for multiple testing were performed.

### Ethical approval

Given the responsibilities set out in the Norwegian Health Research Act, several strands of approval were needed. For the development and implementation of the intervention, we obtained permission by the Norwegian Centre for Research Data (NSD ref: 7435869). Consent in each intervention cluster was obtained from the Head of Municipal Mental Health & Addiction Service, and those participating in qualitative mapping interviews gave individual informed consent. The Regional Research Ethics Committee, who holds authority to grant permission to obtain de-identified specified data without individual consent, considered our procedures to be of minimal risk to individual patients and the study of importance to the patient group, and therefore granted such permission (REC South-East B ref: 267487). Following that, and the approval of a detailed Data Protection Impact Assessment by the Privacy Ombudsman at Akershus University Hospital (ref: 21/08274), our protocol was approved by the Norwegian Patient Registry (NPR ref: S-EA5E432E) before data were released.

## Results

The average rate of involuntary admissions per 10 K adult population in Norwegian municipalities in 2016-17 (inclusion data) was 18.7. As shown in Table 1, both the intervention and control arms had rates above this, 20.3 and 20.6 respectively. The average 18 + population size in the municipalities in the baseline period was 22,637 (min-max 16,959 − 30,269) in the intervention arm and 22,631 (min-max 19,767 − 25,478) in the control arm. The arms were thus comparable at inclusion on population size and the primary outcome. Five invited municipalities declined participation. The municipalities that were randomised but excluded had a mean rate of involuntary admissions of 22.5, and mean 18 + population size of 20,973 (min-max 15,995 − 29,668) and were therefore comparable to those included. In the baseline period, there were more females involuntarily admitted in the control arm, a larger proportion diagnosed with affective disorders (F3), and fewer with schizophrenia spectrum disorders (F2).

**Table 1:** Cluster characteristic at inclusion and involuntary admissions in the baseline period by trial arm

	**Intervention arm**		**Control arm**		**National**	
Municipality population aged ≥ 18 in 2020, mean (min-max)	22,637 (16,959 − 30,269)		22,631 (19,767 − 25,478)			
Involuntary admission rate per 10 K population ≥ 18, 2016/17 (inclusion data)	20.3		20.6		18.7	
**Involuntary admissions, baseline period**						
	**Individuals**	**Events**	**Individuals**	**Events**	**Total sample**	
					**Individuals**	**Events**
N	178	248	169	231	347	479
Male, %	56.2	57.7	48.5	47.2	52.4	52.6
Female, %	43.8	42.3	51.5	52.8	47.6	47.4
Age, mean	45.1		44.6		44.9	
Diagnoses^1,2^, %						
F2: Schizophrenia spectrum	44.9	51.2	40.8	37.2	42.9	44.5
F3: Affective disorders	16.9	14.5	23.7	22.9	20.2	18.6
F1: Addiction disorders	11.2	9.3	10.1	10.0	10.7	9.6
F6: Personality disorders	4.5	5.6	7.1	10.4	5.8	7.9
F4: Neurotic disorders	6.7	6.0	3.6	2.6	5.2	4.4
Other^3^	15.7	13.3	14.8	16.9	15.3	15.0

^1^ Main diagnoses, following the ICD system, associated with the admission. If > 1 diagnosis were recorded, the reported diagnosis is F2 if present, then F3, F1, F6, F4, and ‘other’

^2^ For individuals with more than one admission in the baseline period, the diagnosis associated with their first admission is included

^3^ F0; F5; F7, and; F9

There were a total of 1,562 involuntary admissions during the study period. As shown in Table 2, the change in rate of involuntary admissions from baseline to the intervention period differed with 6.8 admissions per 10 K adults per year between the intervention and control arms (95% CI 1.8 to 11.7; ES 2.7). The difference in change between the baseline period and the post-intervention period was 3.0 (95% CI -3.8 to 9.7; ES 0.9).

**Table 2:** Rate of involuntary admissions per 10,000 adult population per year^1^ in intervention and control arms at different time periods, and comparison of the change in proportions between arms over time, *n* = 1562

	Baseline period	Intervention period	Post-intervention period	Baseline vs. Intervention period	Baseline vs. Post-intervention period
Intervention arm Control arm	21.9 (19.2; 24.6) 20.4 (17.8; 23.0)	20.9 (18.8; 23.1) 26.2 (23.8; 28.6)	23.0 (19.1; 26.9) 24.5 (20.4; 28.5)	-1.0 (-4.5; 2.5) 5.8 (2.2; 9.4)	1.1 (-3.7; 5.9) 4.1 (-0.8; 8.9)
Intervention vs. Control					
mean difference (95% CI) p-value^2^ ES^3^				6.8 (1.8; 11.7) **0.008** 2.7	3.0 (-3.8; 9.7) 0.390 0.9

^1^ Numbers are proportions and are presented with 95% confidence intervals (CI)

^2^ p-values refers to the comparison of change in proportions between arms over time

^3^ ES = Effect Size (Cohen’s d)

In total, 2,542 referrals for involuntary admissions were made during the study period, and 1,405 were confirmed for involuntary status following the second assessment. Table 3 shows that the difference between trial arms regarding changes in referral rates between the baseline and the intervention period was 1.7 (95% CI -4.6 to 8.1; ES 0.5). The difference in changes in the rate of referrals resulting in involuntary status was 1.3 (95% CI -3.4 to 6.0; ES 0.8).

**Table 3:** Rate of referrals for involuntary admissions, and rates of referrals confirmed for involuntary status, per 10,000 population per year^1^ in intervention and control arms at different time periods, and comparison of the change in proportions between arms over time

	Baseline	Intervention period	Baseline vs. Intervention period
***Referrals for involuntary admission (n = 2542)***			
Intervention arm Control arm	31.8 (28.5; 35.1) 35.5 (32.1; 39.0)	36.0 (33.2; 38.8) 41.5 (38.4; 44.5)	4.2 (-0.1; 8.5) 5.9 (1.3; 10.5)
Intervention vs. Control			
mean difference (95% CI) p-value^2^ ES^3^			1.7 (-4.6; 8.1) 0.591 0.5
***Referrals confirmed for involuntary status for involuntary admission***, ***involuntary (n = 1405)***			
Intervention arm Control arm	15.8 (13.5; 18.1) 20.7 (18.0; 23.3)	18.8 (16.7; 20.8) 24.9 (22.5; 27.3)	3.0 (-0.1; 6.1) 4.2 (0.7; 7.8)
Intervention vs. Control			
mean diff. (95% CI) p-value^2^ ES^3^			1.3 (-3.4; 6.0) 0.600 0.8

^1^ Numbers are proportions and are presented with 95% confidence intervals (CI)

^2^ p-values refers to the comparison of change in proportions between arms over time

^3^ ES = Effect Size (Cohen’s d)

When analysing primary outcomes including only those between18-65 years, we observed a difference in change of 6.3 (95% CI 0.3 to 12.3; ES 2.1) between the baseline and intervention periods and 3.6 (95% CI -4.6 to 11.7; ES 0.9) between the baseline and post-intervention periods. The separate analyses of involuntary admissions for observation and treatment showed a difference in change between arms from baseline to the intervention period of 4.1 (95% CI 0.0 to 8.1; ES 2.0) for treatment admissions and 3.4 (95% CI -0.1 to 6.8; ES 1.9) for observation admissions. The same pattern emerged when including only those aged 18–65. Results from the sensitivity analyses are shown in supplementary files.

## Discussion

As far as we have found, this is the first study testing whether an intervention created for and by generalist primary mental health services can reduce local rates of involuntary admissions. The results support our primary hypothesis of lower rates in the intervention arm compared to control between the baseline and intervention periods, with an annual difference of 6.8 in admissions per 10 K population. This difference did not sustain in the post-intervention period when it reduced to 3 admissions per capita, with a wide confidence interval that included the point of no difference, so our hypothesis here was not supported. The same was the case for secondary hypotheses regarding referrals for involuntary admissions and referrals confirmed for involuntary status. The direction of change, favouring the intervention, was the same for all analyses, including sensitivity analyses, and we observed large effect sizes for the difference in admissions (0.9 to 2.7) and moderate for referrals (0.5 to 0.8).

Norway has comparatively high rates of involuntary admissions. A 2018 study, reporting rates of involuntary hospitalised individuals (not events) per 100,000 total population in 22 European countries, reported a median rate of 106.4, with Norway at 150.9 [2]. Measured in events, as we did in our study, the national rate in 2022 was 208 per capita [29]. In that context, our observed difference of 68 admissions per capita, would, should it be replicated in other studies, therefore suggest that targeted and comprehensive interventions at the primary level might have a role to play to achieve policy aims for reduced use of involuntary care set out by governments in Norway [9] and elsewhere [1].

The difference between arms was largely due to an increased rate of involuntary admissions in the control arm between the baseline and intervention periods (from 20.4 to 26.2 per capita), while it remained relatively stable in the intervention arm (from 21.9 to 20.9). Increasing rates is observed in many European countries [2], and in Norway the national rate rose 12% during the study period [29]. This period coincided with the Covid-19 pandemic. Studies investigating the impact of the pandemic on involuntary admissions report a mixed picture with observed increases (e.g. Switzerland), reductions (e.g. Portugal), and stability (e.g. Italy) in rates across countries [30]. In Norway, the observed change was in line with a steady, long-term pattern of year-by-year increases [29], and we have found no analysis of whether this was affected the pandemic. Our results might indicate that the intervention had effect by kerbing the national increase (regardless of what its driver was) during the intervention period.

It might seem counterintuitive that we found a clear and sizeable difference between arms in the change of involuntary admission rates during the intervention period when there was no difference in referrals for such admissions in the same period. This might be explained by how admissions and referrals were measured. Referral analyses did not include CTO revocations (because revocations do not require referrals), but were part of the admission analyses, which is appropriate for our purposes as revocations imply that community care has not helped as intended. The measure of *involuntary admission* therefore encompassed all pathways in, leaving *referrals* a less sensitive indicator of intervention effect, at least in the Norwegian context where national data lack CTO endpoints. Our process evaluation found that introducing systematic use of monitoring data increased municipal services’ awareness of individuals at risk of involuntary admissions. This facilitated other intervention elements, such as targeted support and follow up, closer collaboration between care levels in individual cases, and increased use of municipal crisis beds [28]. As such, we believe it is a reasonable interpretation of our results that services in the intervention arm changed their approach towards those known to be at risk, including those on CTOs, and that this prevented some involuntary admissions. Because of no data on their endpoints, we could not compare prevalence or CTO use between arms to assess this interpretation further, but recommend that associations between rates of involuntary admissions and CTOs should be assessed in future studies.

The shortened post-intervention phase could have exposed observations in that period to random fluctuation. Even so, the lack of difference between arms in the post-intervention period raises the issue of intervention sustainability. The systematic implementation support during the intervention period was reported as a key facilitating factor [28]. This amounted to a structure of regular monitoring meetings and training events initiated by the research team, which enabled intervention sites to share experiences and collaborate in problem solving. These activities also served as reminders to keep focused on the intervention in a context of competing demands [28]. As no support activities were provided in the post-intervention period, the lack of difference might be interpreted as a ‘regression to the mean’ effect once there was less attention towards the intervention’s agenda. Sustaining new ways of working introduced by complex interventions is recognised as difficult, and particularly so in the absence of added resources [14, 31]. Should the limited support provided by the research team be sufficient to sustain the effect of the ReCoN intervention beyond the intervention period, it could prove cost effective at the societal level to allocate time of assigned members of staff to take on this function. This should be tested in future studies.

### Strength and limitations

We used independent, robust data [23] from a national registry, which meant all events of interest were included in the pre-specified analyses. There were no differences between arms at inclusion in primary outcome measures or population size, but some differences in gender and affective disorders associated with admissions. While developing and implementing the intervention in routine services enhanced internal validity [28], the deliberate homogeneity of included municipalities limits generalisation beyond the sample. Moreover, the Zelen’s design, by which intervention but not control municipalities could refuse, might have introduced bias. The excluded municipalities were comparable on population size and admission rates, but refusals meant the level of involuntary admissions in the sample was closer to the national average than anticipated. This could indicate that a stronger effect might be expected in areas with higher rates. Our procedure had the benefit of preventing spill-over effects and ensuring controls were masked [22], but it was impossible to blind the intervention arm. The implementation period coincided with several prolonged Covid-19 lockdowns, which hampered implementation [28]: it is possible that we would have seen different results under more normal circumstances. With an established intervention, it should be possible to apply post-consent randomisation and include ITT analyses in future studies, which should also include fidelity measures to ensure robust implementation assessments [28].

## Conclusion

We found a clear difference between trial arms in our primary outcome of reduced involuntary admissions during the intervention period. Our interpretation is that the intervention curtailed the national trend of raising levels of involuntary admission by more systematically targeting activities and attention towards those identified as being at risk. The effects were not maintained in the post-intervention period, which could mean that prolonged implementation support is necessary.

While our design precludes generalisation beyond the sample, we consider the results as ‘proof of concept’ that primary mental health services, providing general support and collaborating with other local stakeholders, can affect rates of involuntary admissions. Should our primary result be replicated in rigorous studies testing the intervention in more heterogeneous samples and in different contexts, there might be a case for rethinking the distribution of responsibilities, and the accompanying allocation of resources, from secondary to primary mental health care for this area of mental health policy. This would require the availability of adequate resources and capacity to provide good quality community-based primary mental health care, such as that available in our context, as well as the ability, remit and flexibility for services across levels to work collaboratively with the specific aim to reduce the use of involuntary admissions.

## Electronic supplementary material

Below is the link to the electronic supplementary material.

Supplementary Material 1

## Data Availability

No datasets were generated or analysed during the current study.
