# Supplementary material for: Can primary mental health services impact levels of involuntary admissions? A cluster-RCT of the ReCoN intervention
Source: Soc Psychiatry Psychiatr Epidemiol. 2025 Apr 30;60(9):2077–87. doi: 10.1007/s00127-025-02914-3 (PMC12378763; doi:10.1007/s00127-025-02914-3)
Supplement: Supplementary file 1 — Supplementary Material 1 [file 127_2025_2914_MOESM1_ESM.docx]

**Supplementary material**

1. **Systematic literature search for comprehensive intervention developed for generalist primary mental health services with the aim to prevent involuntary admissions.**

Before commencing work on devloping our intervention, we conducted a comprehensive search for literature published between January 1, 1999 and March 19, 2019 in PubMed, PsycINFO, Web of Science as well as the Norwegian Oria, and Tvangsforsk databases and the Scandinavian SveMed+ for studies about the primary mental health service context and involuntary admissions. We found no interventions designed for this care level. We repeated our search in PubMed on May 28, 2024 and again found no new interventions at the primary level. The search terms were as follows:

(((((Schizophrenia) OR (Mental disorders Psychosis)) OR (Severe mental illness)) AND (((((((((((Community mental health services) OR (Local mental health services)) OR (Municipality services)) OR (Primary health care)) OR (Community health services)) OR (General practitioners)) OR (GP)) OR (Outpatient)) OR (After hours care)) OR (Assertive Outreach)) OR (Outreach Programs))) AND ((((((((Involuntary admission) OR (Involuntarily admission)) OR (Involuntary admitted)) OR (Compulsory admission)) OR (Compulsorily admission)) OR (Compulsory admitted)) OR (Coercive admission)) OR (Commitment (psychiatric)))) AND (((((((Intervention) OR (Complex interventions)) OR (Municipal* intervention*)) OR (Community intervention*)) OR (Community service development)) OR (Structural intervention*)) OR (Quality improvement*)).

1. **Sensitivity analyses**

**Supplementary Table 1. Sensitivity analysis including those 18-65 only: Rate of involuntary admissions per 10,000 population per year^1^ in intervention and control arms at different time periods, and comparison between arms in the change in proportions over time, n=1384**

|  | **Baseline period** | **Intervention period** | **Post-intervention period** | **Baseline vs. Intervention period** | **Baseline vs. Post-intervention period** |
| --- | --- | --- | --- | --- | --- |
| **Intervention arm**  **Control arm** | 24.1 (20.9; 27.3)  24.4 (21.1; 27.6) | 23.7 (21.1; 26.2)  30.2 (27.2; 33.2) | 24.6 (20.1; 29.2)  28.4 (23.4; 33.4) | -0.4 (-4.5; 3.7)  5.9 (1.4; 10.3) | 0.5 (-5.0; 6.1)  4.1 (-1.9; 10.1) |
| **Intervention vs. Control**  **mean diff. (95% CI)**  **p-value^2^**  **ES^3^** |  |  |  | 6.3 (0.3; 12.3)  **0.041**  2.1 | 3.6 (-4.6; 11.7)  0.393  0.9 |

^1^ Numbers are proportions and are presented with 95% confidence intervals (CI)

^2^ p-values refer to the comparison of change in proportions between arms over time

^3^ ES= Effect Size (Cohen’s d).

**Supplementary Table 2. Sensitivity analysis including those 18-65 only: Rate of referrals for involuntary admissions, and rates of referrals confirmed for involuntary status, per 10,000 population per year^1^ in intervention and control arms at different time periods, and comparison between arms in the change in proportions over time**

|  | **Baseline period** | **Intervention period** | **Baseline vs. Intervention period** |
| --- | --- | --- | --- |
| ***Referrals for involuntary admission, n=2273*** | | | |
| Intervention arm  Control arm | 35.1 (31.2; 38.9)  42.6 (38.3; 46.9) | 40.7 (37.4; 44.1)  48.5 (44.7; 52.3) | 5.7 (0.6; 10.8)  5.9 (0.2; 11.7) |
| Intervention arm vs. Control arm  mean diff. (95% CI)  p-value^2^  ES^3^ |  | 3.5 | 0.2 (-7.5; 7.9)  0.954  0.1 |
| ***Referrals confirmed for involuntary status, n=1251*** | | | |
| Intervention arm  Control arm | 17.2 (14.5; 20.0)  25.1 (21.7; 28.4) | 21.1 (18.7; 23.5)  28.9 (26.0; 31.8) | 3.9 (0.2; 7.5)  3.9 (-0.6; 8.3) |
| Intervention vs. Control  mean diff. (95% CI)  p-value  **ES^3^** |  |  | 0.0 (-5.7; 5.7)  0.998  0.0 |

^1^ Numbers are proportions and are presented with 95% confidence intervals (CI)

^2^ p-values refers to the comparison of change in proportions between arms over time

^3^ ES= Effect Size (Cohen’s d).

**Supplementary Table 3. Sensitivity analysis separating admissions for observation and treatment: Rate per 10,000 population per year^1^ in intervention and control arms at different time periods, and comparison between arms in the change in proportions over time**

|  | **Baseline period** | **Intervention period** | **Post-intervention period** | **Baseline vs. Intervention period** | **Baseline vs. Post-intervention period** |
| --- | --- | --- | --- | --- | --- |
| ***Involuntary admissions for treatment*** | | | | | |
| Intervention arm  Control arm | 14.2 (12.0; 16.4)  13.3 (11.2; 15.5) | 14.5 (12.7; 16.3)  17.7 (15.7; 19.6) | 16.3 (13.0; 19.6)  14.1 (11.0; 17.1) | 0.3 (-2.6; 3.1)  4.3 (1.4; 7.2) | 2.1 (-1.9; 6.0)  0.7 (-3.0; 4.5) |
| Intervention vs. Control  mean diff. (95% CI)  p-value^2^  **ES^3^** |  |  |  | 4.1 (0.0; 8.1)  0.050  2.0 | -1.3 (-6.8; 4.1)  0.627  -0.5 |
| ***Involuntary admissions for treatment including those 18-65 only*** | | | | | |
| Intervention arm  Control arm | 16.0 (13.4; 18.6)  15.6 (13.0; 18.2) | 16.4 (14.2; 18.5)  20.7 (18.2; 23.2) | .17.5 (13.7; 21.3)  15.6 (11.9; 19.3) | 0.3 (-3.0; 3.7)  5.1 (1.5; 8.7) | 1.5 (-3.1; 6.1)  0.0 (-4.5; 4.6) |
| Intervention vs. Control  mean diff. (95% CI)  p-value  **ES^3^** |  |  |  | 4.8 (-0.2; 9.7)  0.058  2.0 | -1.5 (-7.9; 5.0)  0.658  -0.4 |
| ***Involuntary admissions for observation*** | | | | | |
| Intervention arm  Control arm | 10.4 (8.5; 12.3)  10.4 (8.5; 12.3) | 9.2 (7.8; 10.7)  12.6 (10.9; 14.3) | 10.6 (8.0; 13.3)  14.8 (11.6; 17.9) | -1.2 (-3.5; 1.2)  2.2 (-0.3; 4.7) | 0.2 (-3.0; 3.5)  4.3 (0.7; 8.0) |
| Intervention vs. Control mean diff. (95% CI)  p-value  **ES^3^** |  |  |  | 3.4 (-0.1; 6.8)  0.055  1.9 | 4.1 (-0.8; 9.0)  0.100  1.7 |
| ***Involuntary admissions for observation including those 18-65 only,*** | | | | | |
| Intervention arm  Control arm | 10.8 (8.7; 13.0)  12.2 (9.9; 14.6) | 10.4 (8.7; 12.1)  14.1 (12.1; 16.2) | 10.6 (7.6; 13.6)  17.2 (13.3; 21.1) | -0.5 (-3.2; 2.3)  1.9 (-1.2; 5.0) | -0.2 (-3.9; 3.4)  5.0 (0.4; 9.5) |
| Intervention vs. Control  mean diff. (95% CI)  p-value  **ES^3^** |  |  |  | 2.4 (-1.8; 6.5)  0.259  1.1 | 5.2 (-0.6; 11.0)  0.080  1.8 |

^1^ Numbers are proportions and are presented with 95% confidence intervals (CI)

^2^ p-values refer to the comparison of change in proportions between arms over time

^3^ ES= Effect Size (Cohen’s d).
